# Prediction of *Drosophila melanogaster* gene function using Support Vector Machines

**DOI:** 10.1186/1756-0381-6-8

**Published:** 2013-04-02

**Authors:** Nicholas Mitsakakis, Zak Razak, Michael Escobar, J Timothy Westwood

**Affiliations:** 1 Toronto Health Economics and Technology Assessment (THETA) Collaborative, University of Toronto, Toronto, Canada; 2 Canadian Drosophila Microarray Centre, University of Toronto at Mississauga, Mississauga, Canada; 3 Dalla Lana School of Public Health, University of Toronto, Toronto, Canada; 4 Department of Cell and Systems Biology, University of Toronto at Mississauga, Mississauga, Canada

**Keywords:** Gene ontology, Support Vector Machines, Drosophila melanogaster, Gene expression data, Gene function prediction

## Abstract

**Background:**

While the genomes of hundreds of organisms have been sequenced and good approaches exist for finding protein encoding genes, an important remaining challenge is predicting the functions of the large fraction of genes for which there is no annotation. Large gene expression datasets from microarray experiments already exist and many of these can be used to help assign potential functions to these genes. We have applied Support Vector Machines (SVM), a sigmoid fitting function and a stratified cross‐validation approach to analyze a large microarray experiment dataset from *Drosophila melanogaster* in order to predict possible functions for previously un‐annotated genes. A total of approximately 5043 different genes, or about one‐third of the predicted genes in the *D. melanogaster* genome, are represented in the dataset and 1854 (or 37%) of these genes are un‐annotated.

**Results:**

39 Gene Ontology Biological Process (GO‐BP) categories were found with precision value equal or larger than 0.75, when recall was fixed at the 0.4 level. For two of those categories, we have provided additional support for assigning given genes to the category by showing that the majority of transcripts for the genes belonging in a given category have a similar localization pattern during embryogenesis. Additionally, by assessing the predictions using a confidence score, we have been able to provide a putative GO‐BP term for 1422 previously un‐annotated genes or about 77% of the un‐annotated genes represented on the microarray and about 19% of all of the un‐annotated genes in the *D. melanogaster* genome.

**Conclusions:**

Our study successfully employs a number of SVM classifiers, accompanied by detailed calibration and validation techniques, to generate a number of predictions for new annotations for *D. melanogaster* genes. The applied probabilistic analysis to SVM output improves the interpretability of the prediction results and the objectivity of the validation procedure.

## Background

While the genomes of hundreds of organisms have been sequenced and good approaches exist for finding protein encoding genes, an important remaining challenge is predicting the functions of the large fraction of genes for which there is no annotation. For example, for *Drosophila melanogaster*, approximately 28% of the 14,029 predicted genes have no Gene Ontology (GO) term (either Molecular Function, Biological Process and/or Cellular Component) associated with them (including both curated and electronic annotations) and only 58% have a GO‐BP (Biological Process) term [1]. If one excludes genes that only have an electronic annotation, then only 41% have a GO‐BP term [1]. Functional genomic data and in particular microarray mRNA expression data have been used by numerous researchers as a means to help predict the function of un‐annotated genes. The analysis of such data is based on the premise that genes involved in a particular biological, molecular, and/or biochemical process are often co‐expressed. This co‐expression is dependent on the presence of common cis‐regulatory elements of the co‐regulated genes that bind one or more common transcription factors. A common approach to examine co‐expression profiles from microarray experiment data is to use clustering analysis. In this type of analysis, genes are organized and grouped based on their expression profile, with genes having similar expression pattern being “clustered” (grouped) together. The results of clustering analysis depend (a) on the metric used for comparing the expression profiles of genes that are analyzed and (b) on the clustering algorithm used. Euclidean distance and Pearson’s correlation coefficient are two simple commonly used metrics [2]. Popular clustering algorithms are hierarchical clustering, k‐means clustering and Self Organizing Maps [3].

Although clustering provides an insightful way of exploring gene co‐expression patterns, it does not directly convey any information regarding potential functions of un‐annotated genes. For that purpose, researchers have applied a further layer of analysis to the results of clustering, making use of additional data containing annotations of genes being analyzed. According to this type of analysis, clusters that are “enriched” for a particular gene function (i.e. they contain a larger than by chance number of genes having that function) are “assigned” that function. Subsequently, and following the “guilt‐by‐association” principle, all un‐annotated genes in that cluster are predicted to have the same function [4]‐[7].

In addition to the clustering analyses of genes in an unsupervised fashion and without annotation information, a number of supervised methods have been proposed for predicting gene functions. These methods make use of available information about the annotations of genes in order to discover gene expression patterns that characterize those annotations. Then, functional predictions for un‐annotated genes are made based on how well their expression profiles are matched with “annotated” patterns. Perhaps the most popular class of supervised methods are the binary classifiers, where first, expression data are separated into two classes either having or not having a particular annotation, and then un‐annotated genes are predicted of having or not having the annotation, based on which class their expression profile falls under. Examples of applications of these methods include Support Vector Machines (SVM) [8,9], random forest [10], neural networks [11], factor analysis [12], logistic regression, linear discriminant and quadratic discriminant analysis [13]. The application of the aforementioned approaches for gene function prediction benefits from the use of large data sets where many different experimental treatments or conditions make up the microarray expression dataset (e.g. developmental time points, mutations, specific tissues, environmental conditions, drug treatments, etc.). For the most part, the studies that have tried to do large‐scale gene prediction assignment have used well‐known model organisms where large microarray datasets were available. These include studies in *Saccharomyces cerevisiae*[7,14], *Arabidiopsis thaliana*[13], *D. melanogaster*[15], and *Mus musculus*[9]. Other approaches include assembling and integrating a large number of datasets from different experimental approaches (e.g. microarray expression, genetic interaction, and protein‐protein interaction) to create a network which in turn improves the robustness of the gene function predictions. These include studies in *Saccharomyces cerevisiae*[16], *C. elegans*[17], *D. melanogaster*[10,18] and *Mus musculus*[19].

In this study we focus on the use of SVM for the prediction of *D. melanogaster* gene functions. SVM is a popular machine learning method for classification and regression. Its proven high performance as well as its solid theoretical basis justify its frequent use in many fields, including bioinformatics and predictions of gene functions. As a two‐class classification tool, SVM attempts to separate the data points not in the original feature space but in an “enlarged” higher‐dimensional space instead. The seemingly highly computationally expensive data transformation is not performed but instead, ingeniously, the separation is performed “implicitly” based on their distances measured with the use of a *kernel function*. The SVM algorithm uses optimization techniques to find the surface that gives the optimal margin between the points of the two classes [20,21].

Despite its popularity in bioinformatics along with other research areas, SVM has been used for the prediction of GO‐BP annotations for genes of various organisms (such as *S. cerevisiae*[8] and *M. musculus*[9]) but not for *D. melanogaster*. Our study fills this gap, investigating how well this very popular method works for *D. melanogaster* gene expression data, in particular when the dataset is of a specific structure imposed by the nature of time‐course experiments, as the one we use in our study.

Using microarray data from the life cycle of *D. melanogaster*[22], and a controlled vocabulary for annotation of biological processes associated with *D. melanogaster* genes from the Gene Ontology Consortium (GO‐BP) [23], in this study we propose a method of predicting gene function of un‐annotated genes in the *D. melanogaster* genome by using Support Vector Machines and a two‐level data splitting rotation scheme for validation (double cross‐validation). Our prediction method was evaluated also externally with the use of an independent dataset.

Using this approach we have been able to provide a putative GO‐BP term for about 77% of the un‐annotated genes represented in the dataset and about 19% of all of the un‐annotated genes in the *D. melanogaster* genome helping to bridge the gap for the large number of genes that have little or no annotation. In addition, this SVM approach provides a precision and probability estimate that can help guide users as to the likelihood a given gene belongs to GO annotation class.

## Methods

### Microarray data and annotation sources

The microarray data used in this study was obtained from the series of 138 cDNA microarrays spanning the life cycle of *Drosophila melanogaster*[22]. The microarrays contained probes from 6765 cDNA clones and represented 5043 genes, roughly one third of the total number of genes in the *D. melanogaster* genome. From those genes, 1854 were not annotated with any GO‐BP term at the time of the analysis. Data were obtained from the Stanford Microarray Database [24] and normalized using a ratio‐based method according to the original publication. The dataset can also be obtained from the Gene Expression Omnibus [25], GEO accession number GSE4347. cDNA clone names were converted to primary Flybase Gene Identifiers (FBgn ids) from release 4.2 of the *D. melanogaster* genome using annotation available at Flybase [26]. Biological process annotation was downloaded from the Gene Ontology Consortium [23] in February 2006. Only GO‐BP categories containing a minimum of 10 and a maximum of 999 genes in the dataset were included, and 788 categories met these criteria. Clones with duplicate *computer gene* (CG) numbers were purposely not removed as we were interested in investigating the consistency of the predictions across the duplicates.

### Support Vector Machines

A Support Vector Machine (SVM) is a classification and regression method originally developed by Vapnik [21]. Given a set of *p*‐dimensional vector data *x*_*i*_ and their labels *y*_*i*_ taking the values {-1,+1}, a **linear** Support Vector Machine finds the optimal hyperplane that separates the “positive” from the “negative” class. This plane is maximizing the margin between the two classes. According to the mathematical formulation of the problem, the solution refers to a *weight* vector *w* and a scalar *b* that satisfy the optimization problem

$$\min||w||,y_{i}(w^{T}x_{i}+b)\geq 1,i=1,\ldots ,N,$$

where *N* is the number of samples.

When classes are overlapping and misclassifications are allowed, the above constraints become

$$y_{i}(w^{T}x_{i}+b)\geq 1-\xi_{i},\xi_{i}\geq 0,\sum (\xi_{i})\leq C,$$

where *ξ*_*i*_ are *slack* variables and *C* a constant. Solutions can be generated by using the “Lagrange” formulation of the problem and its “Wolfe dual” problem. Given the solution, the weight vector *w*, for a new point *x*, the function *f*(*x*)=*w*^*T*^*x*+*b* is calculating the discriminant value for *x*, which can be used for its classification to the positive or negative class. It turns out that only a number of training points are important for the determination of the solution, the *support vectors*.

This mechanism can be applied to problems of **non‐linear** separation, after mapping the data onto a higher dimensional space, with the use of a mapping *Φ*. *Φ* is determined by a *kernelK*(.,.) such that *K*(*x*,*y*)=<*Φ*(*x*),*Φ*(*y*)>. The optimization problem is similar with the linear separation case, and following the Lagrange formulation and given a solution of Lagrange multipliers *α*_*i*_, the discriminant function is given by $f(x)=\overset{N_{s}}{\underset{i}{\sum }}\alpha_{i}y_{i}K(x,x_{i})+b$, where *x*_*i*_,*i*=1,…,*N*_*s*_ are the support vectors. It is important to note here that the solution and discriminant function depend on the data only through the kernel, and also that the explicit expression of the mapping *Φ* is not needed for the solutions. This is one of the reasons that make SVM computationally attractive. More information on SVM can be found in [27].

It is evident that SVM depends on the choice of the kernel. After performing a small set of experiments with pilot runs evaluating a number of kernel choices, we decided to use a *radial basis* kernel,

$$K(x,y)=\exp\{||x-y|{|}^{2}/\sigma^{2}\},$$

where $||X||=\sqrt{<x,x>}=\sqrt{x^{T}x}$, over a linear or polynomial kernel. Our decision was also supported by the large popularity of this kernel (previously used, for instance, in [8,9]). We used as *σ* a heuristic value equal to the median value of the distances between positive training points and their nearest negative training points. To combat asymmetric classes, a suggestion from [28] is implemented, where *K*(*x*,*x*) is augmented by *λ*·|*C*|/*N* where |*C*| is the size of the class *C* that *x* belongs to, and *λ* a tuning parameter. For our calculations we use *λ*=*m*/2, where *m* is the median value of the diagonal of the kernel matrix. For the implementation of the SVM algorithm we use the publicly available Gist package [29].

### Estimation of class membership probabilities

For the translation of the discriminant values *f*(*x*) to posterior probabilities of class membership, *p*(*y*=1|*x*), we use the method proposed in [30]. The author proposes a sigmoid function model, where

$$p(y=1|x)=\frac{1}{1+\exp(a\cdot f(x)+b)},$$

with parameters *a*,*b*. The estimation of the parameters *a*,*b* is done by solving a maximum likelihood problem

$$\underset{a,b}{\min}L(a,b),$$

where

$$L(a,b)=\overset{N}{\underset{i=1}{\sum }}(t_{i}\log(p_{i})+(1-t_{i})\log(1-p_{i}))$$

and

$$p_{i}=\frac{1}{1+\exp(a\cdot f_{i}+b)},f_{i}=f(x_{i}),t_{i}=\left\{\begin{array}{cc} \frac{N_{+}+1}{N_{+}+2} & \;\;\text{if}\;y_{i}=1\\ \frac{1}{N_{-}+2} & \;\;\text{if}\;y_{i}=-1 \end{array}\right),i=1,\ldots ,N,$$

where *N*_+_,*N*_-_ are the sizes of the positive and negative class respectively.

For the sigmoid model we use the model fitting algorithm proposed by [31], which is implemented in the Gist package.

### Cross‐validation and performance evaluation

For each selected GO‐BP category a set of annotated genes consisting from the positively annotated and a subset of the negatively annotated of size equal to up to four times the size of the positively annotated is participating in the evaluation of the SVM prediction algorithm. If *I* is the index set of all these genes participating in the cross‐validation, a partition {*I*_1_,*I*_2_,*I*_3_,*I*_4_} is generated, where each *I*_*j*_ has equal size and equal number of positively labelled genes (to the degree that this is achievable). Two of those sets are used as “training set” for the SVM, one set is used for calibration and sigmoid fitting, and the last set is used as “test” set, where probability estimate values are output from SVM and compared with the known annotations of the genes for the evaluation of the method. For each permutation (*α*,*β*,*γ*,*δ*) of {1,2,3,4}, and if gene *g*_*i*_ belongs to set *I*_*α*_, we denote with *p*_*β*|*γ*,*δ*_(*i*) the probability estimate for gene *g*_*i*_ from an SVM classifier that was trained with training set *I*_*γ*_∪*I*_*δ*_ and calibrated with the set *I*_*β*_. Keeping the test set fixed we can have three different ways of constructing the training and calibration sets, and therefore each gene of the test set has three different probability estimate values. For example, for gene *g*_*i*_ in *I*_1_, we have *p*_2|3,4_(*i*),*p*_4|2,3_(*i*) and *p*_3|4,2_(*i*). Those three values are averaged to produce a unique probability estimate for every gene. For each test set (or fold) the probability estimates and true annotations of the genes are used for the calculation of performance measures such as *precision at 20*, *30* and *40*. The values of those measures are then averaged to produced one final measure for the whole SVM procedure. Note here the distinction between the probability estimate generation for each gene that involves averaging over the three possible arrangements of training and calibration sets, and the calculation of the performance measure that is done independently for each one of the four folds (test sets), using each time only the genes of the test set.

For the calculation of precision and recall and the generation of the precision‐recall plots the R package ROCR [32] was used. For the precision‐recall plots, the curves corresponding to the different folds were averaged vertically (i.e. precision values were averaged for the same recall value).

## Results

### Gene function prediction using Support Vector Machines

**Figure 1:**
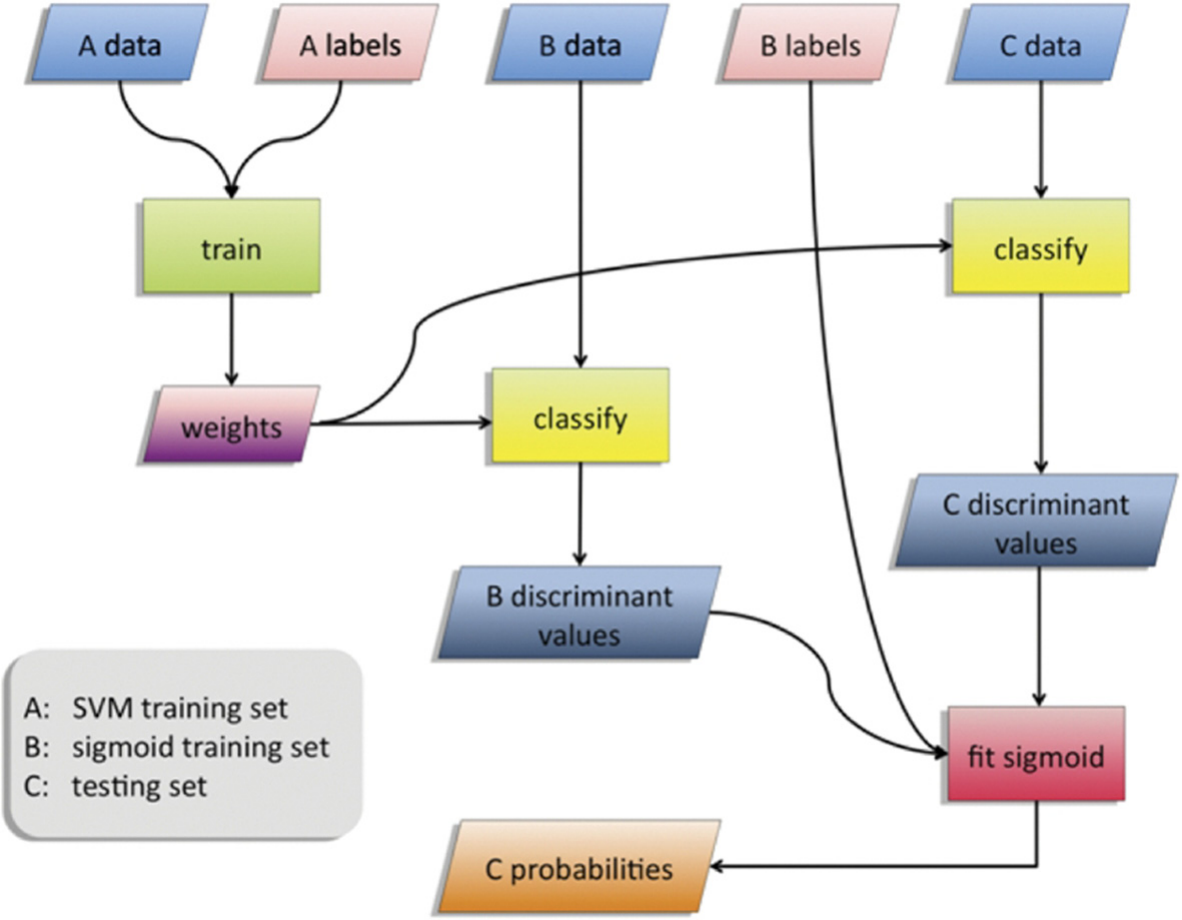
**Flowchart Describing the Probability Estimation Procedure using SVM and the Sigmoid Fitting Function.** First, SVM is trained using dataset A (SVM training set). Then, classification predictions (in the form of discriminant values) for dataset B (sigmoid training or tuning set) are generated. Those predictions along with the known labels of B are used for the fitting of the sigmoid function. Finally, classification results for dataset C (test set) are mapped to estimated class membership probabilities using the fitted sigmoid.

The prediction of gene functions was performed with the use of a number of SVM classifiers. Annotation predictions were made independently for each of 788 GO‐BP categories selected based on their size (see Microarray data and annotation sources in Methods section), based on the results of an SVM classifier. Each SVM was trained with the use of a training set of data points of known labels (in this case, genes with Gene Ontology Biological Processes (GO‐BP) annotation). To remedy any model fitting problem caused by the high imbalance between the positive and negative classes, for every GO‐BP category and SVM classifier we limited the negative examples by randomly selecting a subset of them with a ratio of positive to negative examples being 1:4. The trained system was used for the classification of new, unlabelled data (i.e. prediction of functions for un‐annotated genes). The classification was based on the discriminant value of the data point, which is output by the SVM. As the discriminant value measures the “distance” of the data point from the hyperplane that separates the two classes, traditionally points with discriminant value larger than some threshold (typically zero) are classified as positives and the rest as negatives. Here, we refrain from translating the discriminant values to binary classifications, but, instead, we used a previously published algorithm to estimate the posterior probability of a gene membership to each GO‐BP category. The algorithm fits a sigmoid function to the discriminant values of labeled data and it uses this function to approximate posterior class probabilities for unlabelled data [31]. A flowchart describing the probability estimation procedure using SVM and the sigmoid fitting function is shown in Figure 1.

We validated the performance of SVM and sigmoid fitting function with the use of stratified cross‐validation, where the initial set of known data points (positively and selected negatively annotated genes) was randomly partitioned into four equal size sets, with equal number of positively annotated genes [33]. For every possible set combination, two fourths were used as “SVM‐training” set (used to train the SVM classifier), one fourth as a “sigmoid‐training” set (used for fitting the sigmoid function to the discriminant values) and one fourth as an overall “test” set, for which the membership probabilities were estimated. Predictions results for the latter set can then be validated, since the true labels (gene functions) are known. For more details in the cross‐validation procedure used see Methods section.

For any chosen probability threshold value *p*_*t*_ we can calculate the corresponding *precision* value, defined as the ratio of the number of genes that have average probability estimate equal or larger than *p*_*t*_ and belong to the class, divided by the number of genes that have a probability estimate equal or larger than *p*_*t*_ (i.e. True Positives / Predicted Positives). Similarly, *recall* is defined as the proportion of the genes annotated with the term that also have average probability estimate equal or larger than the threshold (i.e. True Positives / Total Positives). While precision gives a measure of the accuracy of the prediction, recall measures the coverage or completeness. The prediction performance of SVM for a specific GO‐BP category was evaluated with the use of a measure equal to the precision value corresponding to a specific recall value (e.g. 20%, 30%, 40%). Precision at 20, 30 and 40 were calculated for each fold and averaged over all four folds, and the number of categories with high performance measure values were calculated. More specifically, the method identified 39 high‐precision categories reaching precision at 40 values equal or larger than 0.75. Table 1 presents these categories. Some of these categories are related through ancestor‐predecessor relationships and therefore have a certain amount of redundancy. (The relationship between the categories is shown in a GO graph presented in Additional file 1: Figure S1.) Taking into account these relationships as well as the size of the categories, we came up with a subset of 24 categories of minimum redundancy.

**Table 1:** Selected GO‐BP categories with high precision values

**Category names**	**Size**	**Max‐prec‐40**
Detection of external stimulus GO 0009581	36	1.000
**Detection of light stimulus GO 0009583**	28	1.000
Rhodopsin mediated phototransduction GO 0009586	14	1.000
Detection of stimulus GO 0051606	38	0.964
**Chitin metabolism GO 0006030**	25	0.958
**Regulation of mitosis GO 0007088**	21	0.938
Phototransduction GO 0007602	25	0.938
**Fatty acid oxidation GO 0019395**	20	0.929
**Oxidative phosphorylation GO 0006119**	68	0.919
ATP synthesis coupled electron transport sensu Eukaryota GO 0042775	22	0.917
**Cell matrix adhesion GO 0007160**	25	0.909
ATP synthesis coupled electron transport GO 0042773	22	0.906
**Segment specification GO 0007379**	21	0.900
**Interphase of mitotic cell cycle GO 0051329**	16	0.900
Cell substrate adhesion GO 0031589	25	0.875
**Homophilic cell adhesion GO 0007156**	16	0.854
**Regulation of exocytosis GO 0017157**	20	0.850
Aerobic respiration GO 0009060	31	0.845
Response to light stimulus GO 0009416	33	0.839
**Chromosome condensation GO 0030261**	27	0.833
**Sphingolipid metabolism GO 0006665**	12	0.833
**Neuromuscular junction development GO 0007528**	19	0.833
**Chromatin assembly GO 0031497**	20	0.833
**Nuclear import GO 0051170**	33	0.817
Glucosamine metabolism GO 0006041	27	0.813
N acetylglucosamine metabolism GO 0006044	27	0.813
SRP dependent cotranslational protein targeting to membrane GO 0006614	12	0.813
**RNA transport GO 0050658**	14	0.800
**Membrane lipid biosynthesis GO 0046467**	20	0.792
DNA amplification GO 0006277	19	0.788
**Chorion gene amplification GO 0007307**	18	0.783
**DNA dependent DNA replication GO 0006261**	53	0.776
response to radiation GO 0009314	37	0.774
**Ribosome biogenesis GO 0007046**	18	0.771
**Regulation of DNA metabolism GO 0051052**	13	0.771
**Ventral cord development GO 0007419**	14	0.767
Protein targeting to membrane GO 0006612	12	0.750
**Neuron recognition GO 0008038**	12	0.750
**Protein targeting to ER GO 0045047**	12	0.750

Each one of these 39 categories has a fold‐average precision‐at‐40 (i.e. that corresponds to recall value = 0.4) equal or larger than 0.75. The number of genes in the dataset annotated with a category is also shown. Bold fonts are used for the 24 GO‐BP categories that show minimal redundancy with genes found in other categories.

**Figure 2:**
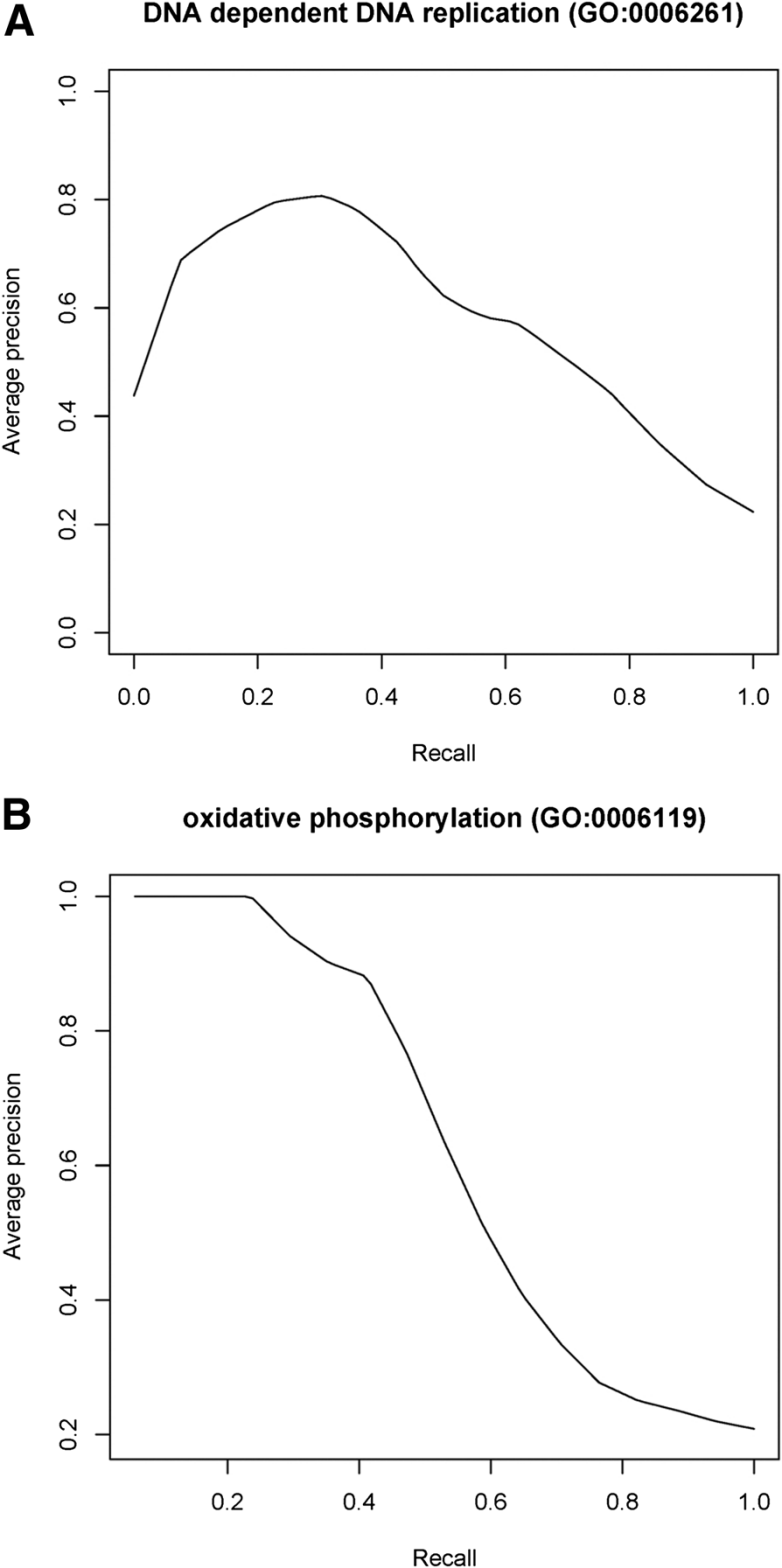
**Precision‐recall Plots for Two of the GO‐ Biological Process Categories.** Plots show the fold‐average precision that corresponds to a recall value for (**A**) DNA‐dependent DNA replication, and (**B**) oxidative phosphorylation GO categories. Vertical averaging method was used.

Two of the high‐precision GO‐BP categories, *DNA‐dependent DNA replication* and *oxidative phosphorylation*, were chosen for further analysis. Precision‐recall plots were generated for both categories and presented in Figures 2A and 2B.

**Figure 3:**
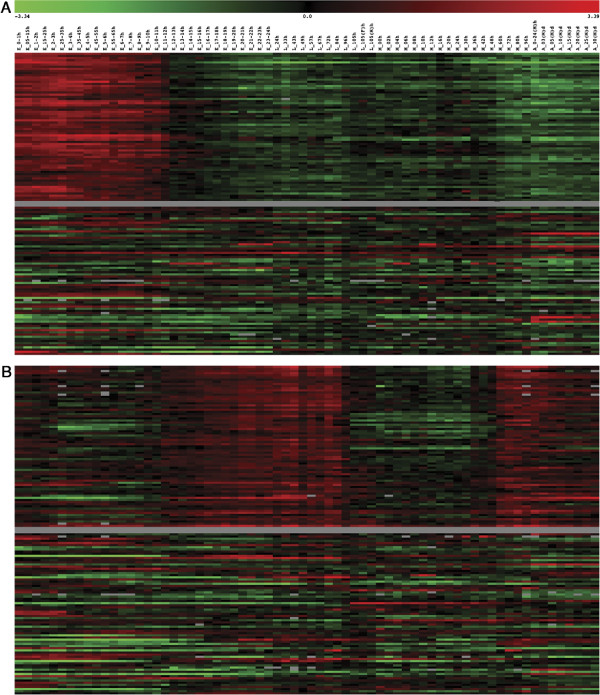
**Developmental Transcription Profiles for Two of the GO‐ Biological Process Categories.** The gene expression profiles of two of the GO‐BP categories: (**A**) DNA‐dependent DNA replication, and (**B**) oxidative phosphorylation are shown. The top portion of each of the category figures contains the expression profiles throughout development for each gene in the category in the same order as they appear in either Table 2 or 3 respectively. The bottom portion of each category represents an equal number of genes randomly selected from the expression profiles in the entire data set. Red denotes genes having up‐regulated transcription at a given time point and green down‐regulated genes. The scale at the top of the figure indicates the degree of up‐ (in red) and down‐ (in green) regulation (in fold change).

Developmental gene expression profiles for the genes in these two categories are shown in Figure 3. For all of the expression profiles, the gene expression ratio for a given gene at a given time point is calculated by dividing the intensity signal for that gene at that time point by the average intensity for that gene throughout development. For the genes belonging to the DNA‐dependent DNA replication category, there is a strong up‐regulation of these transcripts from the earliest time points in development (0.5‐1.5h embryos) through approximately mid‐embryogenesis (11‐12h embryos) (Figure 3A). By way of comparison, the gene expression profiles of an equivalent number of genes selected at random are shown in the lower half of the figure and in general, the pattern seen for the DNA‐dependent DNA replication genes is not observed for other genes. The genes belonging to the oxidative phosphorylation category have a very different transcriptional profile. They are generally expressed at low levels during early embryogenesis and begin to show increased expression beginning in late embryogenesis and throughout the three larval stages. At late third instar (L 96h and 105h), prepupal and the majority of pupal stages (M00h‐M48h) these genes are down‐regulated and then are dramatically up‐regulated in late metamorphosis (M60h) and stay high until early adulthood (Figure 3B). It should be noted that during embryogenesis and metamorphosis the organisms are not feeding or moving but during the larval and adult stages they are. Interestingly, the oxidative phorphorylation genes are up‐regulated well in advance of the organisms moving or feeding, suggesting that the organism is preparing itself for the next stage of the development that will require the enzyme activities to carry out this process.

**Table 2:** Gene Lists and Fluorescent In Situ Hybridization (FISH) analysis for DNA‐dependent DNA replication GO‐BP category

**GeneID**	**Sample**	**FISH**	**Gene**	**Prob.**	**GeneID**	**Sample**	**FISH**	**Gene**	**Prob.**
		**match**	**prec.**	**score**			**match**	**prec.**	**score**
CG5924	LD38710		0.923	0.705	CG1109	LD26389		0.798	0.592
CG1109	LD27350		0.923	0.736	CG8290	LD37351	+	0.798	0.575
CG7663	LD46979	+	0.923	0.724	CG2910	GH11110		0.798	0.598
CG7384	LD46023		0.923	0.76	CG10364	LD32040	-	0.798	0.58
CG14464	LD29015		0.923	0.707	CG5877	LD29352		0.79	0.572
CG16892	LD26813	+	0.923	0.728	CG10625	LD39545	-	0.79	0.568
CG1578	LD28359	+	0.923	0.755	CG17509	GH12788		0.787	0.568
CG16892	LD42637		0.923	0.739	CG11409	LD40802		0.787	0.607
CG11122	LD29040		0.86	0.644	CG30007	LD29335		0.787	0.58
CG9300	LD21924		0.86	0.666	CG17681	LD30009		0.787	0.573
CG3287	SD03445		0.86	0.643	CG18622	LD26416		0.787	0.539
CG1960	GH21591		0.86	0.66	CG31152	LD29477	+	0.787	0.545
CG1024	LD28076		0.86	0.652	CG11990	LD47989	+	0.787	0.549
CG13096	SD03546		0.86	0.664	CG6724	LD40657		0.783	0.589
CG11596	LD45925		0.86	0.682	CG32069	LD47413		0.783	0.565
CG4857	LD29423	+	0.86	0.638	CG2962	LD27487		0.783	0.578
CG4949	LD46305	+	0.86	0.669	CG6049	LD27763		0.776	0.558
CG11943	SD04935		0.86	0.703	CG2260	LD30339		0.772	0.563
CG2469	LD30285	+	0.86	0.677	CG3735	LD35854		0.771	0.553
CG11596	LD42227	+	0.839	0.66	CG7110	LD39933	-	0.771	0.577
CG12785	LD27528		0.839	0.616	CG12202	LD30511	+	0.771	0.584
CG11329	LD26217		0.839	0.619	CG9591	LD26057	+	0.771	0.554
CG6066	LD27582		0.839	0.621	CG12340	LD26050		0.771	0.552
CG17050	LD35611		0.839	0.647	CG30020	LD40262		0.771	0.549
CG18004	LD27741		0.839	0.662	CG12050	LD30416	+	0.771	0.561
CG1647	LD30287		0.839	0.591	CG6151	LD28933		0.766	0.567
CG31697	SD02518		0.839	0.618	CG14657	LD28447		0.766	0.556
CG15736	LD33780	+	0.83	0.619	CG4203	LD29184		0.761	0.537
CG2691	LD46946		0.829	0.61	CG4281	SD03946		0.76	0.548
CG7728	LD39680	+	0.812	0.592	CG14005	LD30293		0.76	0.566
CG31163	SD09611	+	0.812	0.598	CG9028	LD27194	+	0.76	0.555
CG3680	LD27862		0.81	0.63	CG7824	LD26655		0.76	0.542
CG3362	LD28544		0.81	0.595	CG7407	LD29166	-	0.758	0.548
NA ^∗^	LD42550		0.81	0.612	CG3338	LD27356	+	0.756	0.524
CG11906	LD27134		0.798	0.566					

^∗^CG number not known

DNA‐dependent DNA replication GO‐BP category (along with oxidative phosphorylation) was selected for further biological validation using fluorescent in situ hybridization (FISH) analysis of early *D. melanogaster* embryos. In this table, genes that are predicted to belong to this category are shown along with information regarding their presence in the FISH database. Each of the genes (i.e. CG numbers) in the list was searched in a *D. melanogaster* FISH database to visually examine the spatial and temporal mRNA expression for that gene during early embryogenesis. Genes that are marked with (+) or (-) sign had images present in the database. Those that had patterns that largely matched that of known genes in the category are marked with a (+). If either their temporal or their spatial pattern did not match the known gene pattern, they are marked with (-). For each gene the “gene‐precision” score and the average probability estimate output from SVM are reported.

**Table 3:** Gene Lists and Fluorescent In Situ Hybridization (FISH) analysis for oxidative phosphorylation GO category

**GeneID**	**Sample**	**FISH**	**Gene**	**Prob.**	**GeneID**	**Sample**	**FISH**	**Gene**	**Prob.**
		**match**	**prec.**	**score**			**match**	**prec.**	**score**
CG12934	LP05346		1	0.814	CG5608	LD32461		0.898	0.537
CG1715	LD33960	+	1	0.81	CG8401	GH01937		0.897	0.548
CG33316	SD08735		0.975	0.656	CG6094	GH26345		0.897	0.553
CG5523	GH14535		0.975	0.754	CG9813	GH04365		0.892	0.522
CG10675	GH14673		0.975	0.79	CG13220	GH06079	+	0.885	0.538
CG9921	GH07174		0.975	0.714	CG9056	GH11503		0.885	0.559
CG8486	GH04578		0.975	0.594	CG4577	GH23863		0.875	0.535
CG8086	GH25625		0.975	0.752	CG5325	GM14611		0.871	0.539
CG10075	GH25609	+	0.975	0.679	CG7710	LP03578		0.871	0.544
CG30116	GH04922		0.975	0.677	CG14125	GH07601		0.868	0.502
CG5532	GH01442	+	0.975	0.662	CG1859	GH26443		0.868	0.52
CG12239	GH14380		0.975	0.66	CG5325	GH03076		0.829	0.505
CG8740	GH05582	+	0.975	0.658	CG1135	GH01794		0.829	0.489
CG18616	GH04932	+	0.975	0.737	CG4757	SD01814		0.826	0.496
CG3420	GH11502	+	0.975	0.682	CG5989	GH26459	+	0.826	0.481
CG15669	GH02495		0.975	0.629	CG3153	GH04701		0.812	0.468
CG11203	GH26638		0.975	0.691	CG1927	GH11112		0.812	0.462
CG6044	GH12587		0.975	0.61	CG7217	LD45324		0.812	0.457
CG5903	GH13386	-	0.975	0.768	CG6123	GH13094		0.812	0.47
CG14823	GH02020		0.975	0.656	CG2269	GH06015	-	0.809	0.48
CG13367	GH14959		0.975	0.7	CG4589	LP05955		0.807	0.489
CG6424	GH08256		0.975	0.743	CG14438	GH25521		0.8	0.47
CG7083	GH27162		0.947	0.68	CG8206	GH04557		0.794	0.437
CG3631	LD29155	+	0.925	0.655	CG9336	GH22472		0.794	0.453
CG4281	GH10944	+	0.925	0.604	CG7570	GH27163		0.794	0.459
CG12706	GH14695		0.925	0.648	CG10973	LD28549		0.792	0.452
CG10249	GH11802		0.925	0.56	CG7506	GH02466		0.788	0.467
CG14292	GH14813		0.912	0.592	CG6455	GH04666		0.786	0.463
CG4972	GH14975		0.912	0.592	CG17828	GH04984	+	0.778	0.432
CG32795	HL08104	-	0.912	0.532	CG15765	GH28601		0.778	0.447
CG4975	GH18454		0.912	0.638	CG10585	GH23839	+	0.775	0.432
CG6550	GH28477	+	0.912	0.609	CG10039	GH11404		0.775	0.436
CG3971	GH11554	+	0.912	0.549	CG14817	GH01621		0.771	0.433
CG6659	LD45943	+	0.912	0.578	CG17666	GH08313		0.762	0.42
CG15386	GH19557		0.906	0.516	CG11737	GH22337	+	0.761	0.439
CG1231	GH01151		0.906	0.582	CG7358	GH14795		0.759	0.436
CG15067	GH14961		0.906	0.545	CG5773	GH07612		0.758	0.432
CG6008	GH05862		0.898	0.557					

This table contains the genes that were predicted to belong to oxidative phospholylation GO‐BP category along with information regarding the presence in the FISH database. Notation is similar to Table 2.

### Gene function predictions for un‐annotated genes

One approach of declaring an un‐annotated gene as predicted to be annotated with some GO‐BP term, is to ascertain if its probability estimate output from the SVM specific for this term was larger than some threshold value (e.g. 0.5) and then assign the un‐annotated gene with that term. However, drawbacks to this approach include the arbitrary choice of the threshold and the fact that it ignores the evaluation of the performance of the classifier, as measured by cross‐validation. In this approach, any gene that has probability estimate score larger than 0.5 (or any other pre‐specified threshold value) for two categories A and B, will be predicted to belong to both A and B, regardless how well SVM is performing for these categories. A third problem with this method is that it assumes that probability estimate values are comparable between different GO‐BP categories. Although the purpose of the sigmoid function fit method is to provide with comparable estimates, one cannot be certain that this is necessarily obtained.

We have chosen an approach that takes into account the predictive behaviour of SVM for a specific GO‐BP term, when a prediction is made for an un‐annotated gene, as well as it addresses the other issues mentioned above. For each gene‐GO category pair a “gene precision” score is assigned to be equal to the maximum precision that can be achieved using thresholds smaller or equal to the probability estimate of the gene (output from SVM). If the gene is to be included in the “positive” predictions from SVM (and therefore to be assigned the GO‐BP function), the threshold has to be such that it will give a precision value for the classifier not larger than the “gene precision” score for the gene‐GO category pair. This score measures how probable is for the gene to truly belong to GO‐BP, given its probability estimate output from SVM, and also given the precision value of SVM for the particular category. This gene precision score is calculated separately for each one of the four folds (since for each fold probability estimates are generated for all un‐annotated genes) and the four fold‐specific scores are averaged.

**Figure 4:**
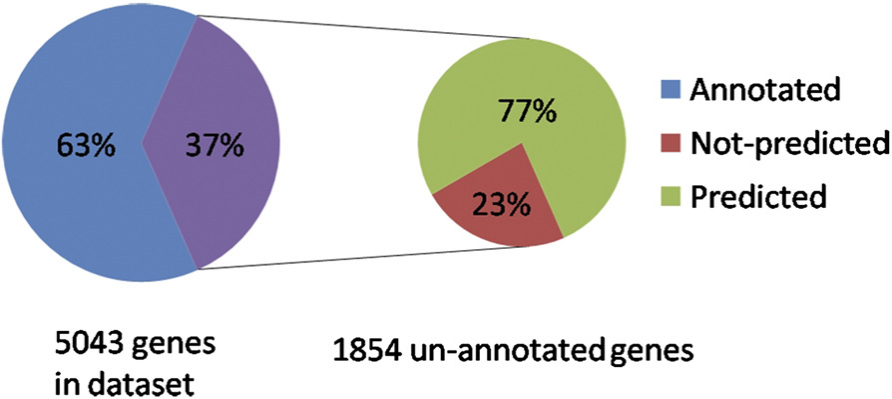
**The Proportion of genes that are Annotated, Un‐annotated, and Predicted by the SVM to Belong to a GO‐BP Category.** Genes from the developmental time course data set that had no GO‐BP annotation were broken down into genes that were predicted with high confidence to belong to a GO‐BP category (predicted), and those that had low prediction values (not predicted).

We can then declare as gene function predictions all the gene‐category pairs that have “gene precision” score equal or larger than some threshold value. More specifically, 9887 new predictions representing 1422 genes (CG numbers) were generated corresponding to a gene precision score larger than 0.75. A total of approximately 5043 different genes, or about one‐third of the predicted genes in the *D. melanogaster* genome, are represented in the dataset. 1854 (or 37%) of these genes are un‐annotated and therefore this method is providing gene function predictions for about 77% (1422/1854) of the un‐annotated genes represented on the microarray and about 19% of all of the un‐annotated genes in the *D. melanogaster* genome. A graphical representation of these results is shown in Figure 4. The complete list of the these predictions can be found in Additional file 2: Table S1.

### Experimental support of predicted gene functions

The un‐annotated genes contained in two of the high‐precision GO‐BP categories, DNA‐dependent DNA replication and oxidative phosphorylation are listed in Tables 2 and 3. For each gene, the gene precision score is indicated. The transcripts for genes involved in the same function are often co‐localized within cells. Each of the genes (i.e. CG numbers) on each list was searched in a *D. melanogaster* FISH database [34,35] to visually examine the spatial and temporal mRNA expression for that gene during early embryogenesis. Genes that are marked with a (+) or (-) had images present in the database. For the genes with images present in the FISH database, their spatial and temporal pattern were compared with those of known genes annotated with the category. Genes that had patterns that largely matched that of known genes in the category are marked with a (+). If either their temporal or their spatial pattern did not match the known gene pattern, they are marked with (-).

**Figure 5:**
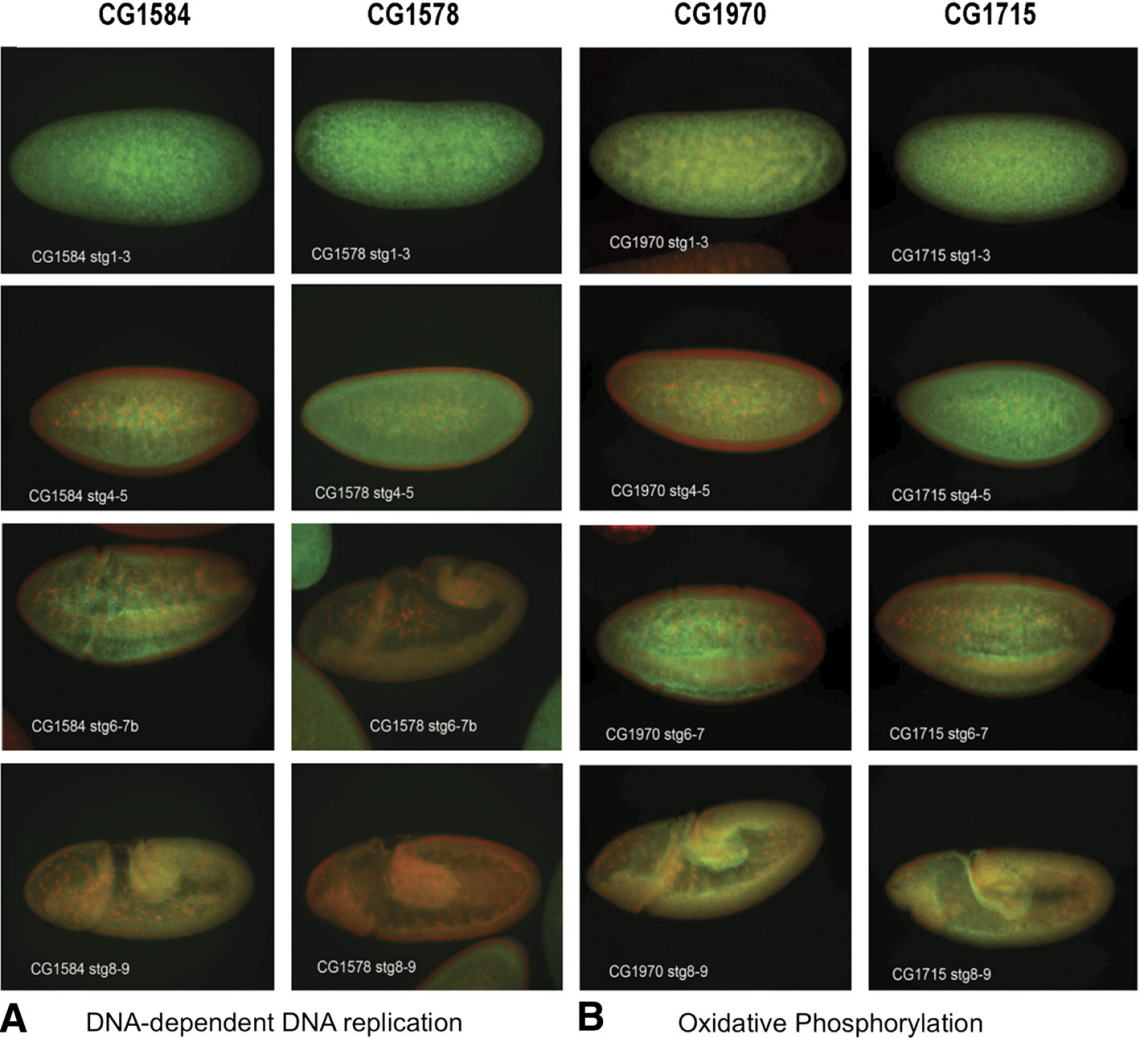
**Fluorescent In Situ Hybridization (FISH) Images of Annotated and Un‐annotated Gene mRNAs.** One annotated gene and one un‐annotated gene from each of the two GO‐BP categories shown in Tables 2 and 3 were chosen and FISH images for those genes was retrieved from the FlyFISH database. Four different stage categories of early embryogenesis are shown. Green fluorescence represents the mRNA localization pattern for that transcript/gene and red fluorescence is showing the position of nuclei within the organism at that stage. CG1584 = Orc6, Origin recognition complex subunit 6. Its molecular function is described as DNA binding and it is involved in the biological processes: DNA replication initiation; DNA‐dependent DNA replication; chromatin silencing. CG1970‐ NADH dehydrogenase (ubiquinone) activity. It is involved in the biological process mitochondrial electron transport.

One annotated gene from the dataset known to belong to the GO‐BP and one un‐annotated gene from each of the two GO‐BP categories listed in each of Tables 2 and 3 were chosen and FISH images for those genes was retrieved from the FlyFISH database. Four different stage categories of early embryogenesis are shown in Figure 5. Green fluorescence represents the mRNA localization pattern for that transcript/gene and red fluorescence is showing the position of nuclei within the organism at that stage. For the known gene belonging to the GO‐BP DNA‐dependent DNA replication, the annotation for gene CG1584 (Gene name = Orc6, i.e. Origin recognition complex subunit 6) has a molecular function described as DNA binding. It is involved in the following biological processes: DNA replication initiation; DNA‐dependent DNA replication; chromatin silencing. The un‐annotated gene, CG1578 (from Table 2), had a very similar in situ hybridization pattern (Figure 5A). For the oxidative phophorylation GO‐BP category, the known gene is CG1970, annotated as having NADH dehydrogenase (ubiquinone) activity. It is involved in the biological process mitochondrial electron transport. The un‐annotated gene, CG1715 (from Table 3), has an in situ hybridization pattern closely resembling the pattern seen for CG1970.

Of the un‐annotated genes that are predicted to belong to the DNA‐dependent DNA‐replication GO‐BP genes, 22 had images in the FlyFISH database and 18 out of 22 (82%) had FISH images that were the same or very similar to the known genes belonging to the category (Table 2). Similarly, of the un‐annotated genes that are predicted to belong to the oxidative phosphorylation GO‐BP category, 19 had images in the FlyFISH database and 16 out of 19 (84%) had FISH images that were the same or very similar to the known genes belonging to the category (Table 3).

## Discussion

We have applied Support Vector Machines (SVM) and a stratified cross‐validation approach to analyze a large microarray experiment dataset from *D. melanogaster* in order to predict possible functions for previously un‐annotated genes. This approach successfully generated a preliminary GO Biological Process annotation for a large number of these genes.

The SVM analysis, employing a sigmoid fitting function, generated annotation probability values for all the genes in the dataset. Using a recall value of 40%, 39 high‐precision GO‐BP categories were identified and 77% of the un‐annotated genes in the dataset have been assigned to one or more of these GO‐BP categories. Assigning new annotations based on gene‐precision scores takes into account both the overall precision of the GO‐BP category, as well as the individual probability of the gene, outcome of SVM and the sigmoid function. In that way, gene predictions of high confidence can be considered even for GO‐BP categories that have an overall poor precision performance.

We chose two of the high‐precision GO‐BP categories to validate using an independent data set (the FlyFISH database). These two categories, DNA‐dependent DNA replication and oxidative phosphorylation, were selected for a number of reasons but chiefly because each contained a fairly large number of previously unannotated genes allowing for a larger number of genes that could be cross‐checked in the FlyFISH database and that the GO‐BP description was fairly precise allowing for the potential to examine whether the unannotated genes are actually involved in the biological process they had been assigned to. While it could be argued that the use of FlyFISH data is perhaps too similar of a data type to be useful (i.e. is also gene transcription data), our main goal was to use data that: was from a completely independent source; had at least some data that differed in type from the original microarray data (in this case spatial data in addition to temporal data); where the raw data could be inspected independently at the gene level so that any computer or user assigned functional classification of the genes would not bias the validation. Given the high degree of precision that some of the unannotated genes have for belonging to a specific GO‐BP category, especially for the oxidative phosphorylation category, we feel quite confident that a large number of these genes will be shown in the future to be directly or indirectly involved in these biological processes. Gene expression pattern data has been used in the past to uncover the function of a gene. In a study by Hughes and coworkers [14], a large compendium of yeast gene expression data was examined for co‐expression patterns using hierarchical clustering and among several findings, they discovered that a previously unannotated gene, YER044c (now ERG28), had a gene expression pattern that was highly similar to genes that were known to be involved in ergosterol biosynthesis. Further biochemical and genetic experiments supported that the gene was indeed involved in this process [14].

In the original Arbeitman and coworkers study [22], the authors performed different hierarchical clustering analyses to identify groups of genes that were temporally co‐regulated and/or restricted to specific biological sample type (e.g. mutant or sex). The authors also identified several GO classes that could be assigned to subsets of the co‐regulated genes. While many of the GO classes reported were fairly low (i.e. general) in the GO annotation hierarchy (e.g. enzyme, metabolism) others were more detailed. Five of the more detailed GO categories reported by Arbeitman and coworkers [22] overlap with the 39 GO biological categories identified in this study including: mitochondria/electron transport; ribosome; cell cycle; cell adhesion; and phototransduction.

We attempted to compare our results with those from the study of Yan and co‐workers [10], who also assigned GO terms to groups of similarly behaving genes to a dataset which in part included the data we used in our study. The authors of that study apply a different computational method (Random Forest) to a number of data sources (including gene expression data) for the prediction of GO‐BP annotations. For the comparison with our study, first, predictions based on expression data only have to be extracted and, subsequently, precision and recall measures need to be calculated using methods similar to those used in our study. There were a few issues that made the comparison between the two studies difficult. First, Yan and co‐workers do not apply stratification in their 10‐fold cross‐validation. As a result, for some of the GO‐BP categories (and the associated classifiers) there are a number of folds that contain only negative classes, and therefore classification (and calculation of precision) cannot be performed. Estimates of measures such as precision at 40 could be generated based on the remaining folds, but this practice is generally not recommended as it may introduce bias [33]. Second, the number of categories that are represented in both studies (273) is much smaller than those that are represented in each study individually. If we exclude those that give folds without positively labeled representatives, we are left out with a very small number of GO‐BP categories for comparison. Due to these issues a strict comparison of the results from the two studies is not particularly valuable. For reference purposes we mention here that in Yan et al. study, 8 GO‐BP categories gave a precision at 40 value equal or larger than 0.75 after excluding the GO terms with less than 5 folds representing both classes, while the number of selected categories dropped to 1 if we exclude those with less than 8 folds representing both classes. Also, the reported new predictions in Yan et al. involve 2062 genes at the confidence level of 0.2, 213 genes at the confidence level of 0.5 and 11 genes at the confidence level of 0.75. It should be noted, though, that the confidence level is Yan et al. study was computed differently from the gene precision score in our study and the two are not necessarily comparable.

We believe that the methods used in this study can be applied to other microarray and RNA‐seq datasets. Our own experience trying this approach on other data suggests that the robustness of the results is largely dependent on both the number of array experiments that are in the dataset as well as on the type of experiment(s) the data represent. In this case, a developmental time course with numerous time points provided the ability to identify a large number of co‐expressed genes.

## Conclusions

In this study we have applied Support Vector Machines for the prediction of GO‐BP annotation terms for *D. melanogaster* genes, using published time‐course gene expression data. We have assessed the predictive ability of the method using an elaborate stratified double cross‐validation procedure, involving the fitting of a sigmoid function on the raw output from SVM. Precision and recall values for each GO‐BP function were calculated, describing in detail the performance of the prediction. On a second level, our results were validated externally using independent data from FlyFISH database. Finally, we applied SVM for the prediction of GO‐BP annotations of un‐annotated genes, providing certainty estimates for those predictions, based on a method that uses the results from the cross‐validation and the predictive performance of SVM for the specific GO‐BP category.

We believe that this is the first study that investigates the performance of SVM for GO‐BP prediction for *D. melanogaster* genes and provides a comprehensive list of new annotation predictions that can be further researched experimentally. We believe that our study is of great value to researchers interested in computational annotation of genes, as well as to the community of *D. melanogaster* researchers.

## Abbreviations

GO‐BP: Gene Ontology Biological Processes; SVM: Support Vector Machine

## Competing interests

The authors declare that they have no competing interests.

## Authors’ contributions

NM participated in the conception and design of the study, performed the analysis of the data, participated in the creation of the figures and tables and wrote part of the manuscript. ZR pre‐processed the data and participated in the creation of the figures and tables. ME participated in the conception and design of the study and revised the manuscript for critically important content. TW participated in the conception and design of the study, participated in the creation of figures and tables, wrote part of the manuscript and revised the manuscript for critically important content. All authors read and approved the final manuscript.

## Supplementary Material

Additional file 1 **The Parent‐Child Relationship Between Ontology Biological Process Categories.** Figure showing a joined Gene Ontology graph providing an overview of the relationship between the 39 Gene Ontology Biological Process (GO‐BP) categories that were identified from the output of the SVM as having a precision‐at‐40 value equal or larger than 0.75, as indicated in the highlighted polygonal nodes. Click here for file

Additional file 2 **Selected GO‐BP Predictions for Previously Unannotated Genes.** Table showing all new predictions with average gene‐precision score equal or larger than 0.75. Detailed information on gene‐precision scores and probability estimates for each one of the four folds is shown. Click here for file
