# Supplementary figures and images for: Prediction of Drosophila melanogaster gene function using Support Vector Machines
Source: BioData Min. 2013 Apr 2;6:8. doi: 10.1186/1756-0381-6-8 (PMC3669044; doi:10.1186/1756-0381-6-8)

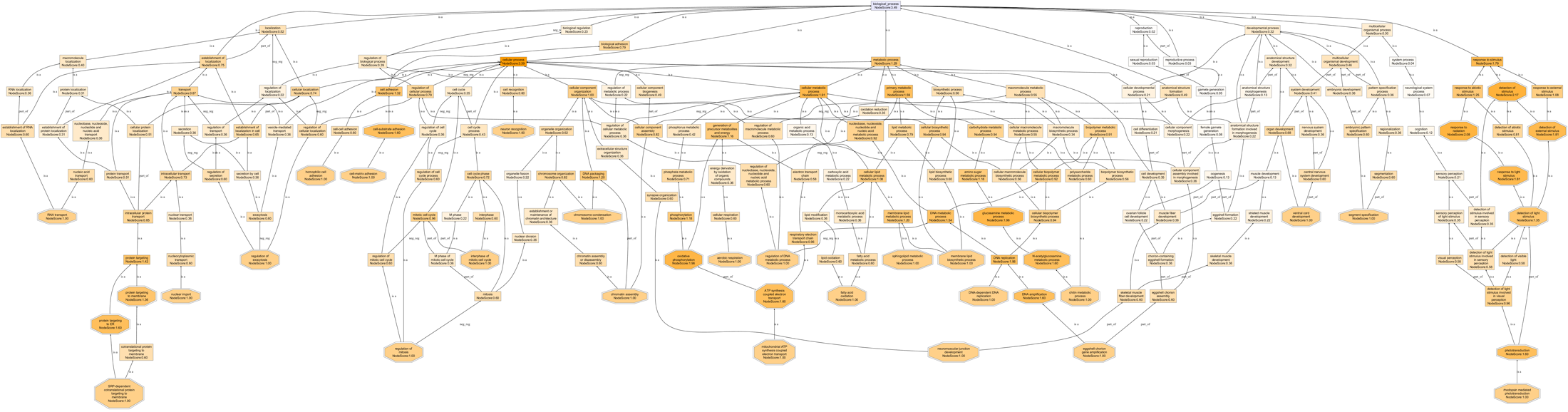

Supplement: Additional file 1 — The Parent‐Child Relationship Between Ontology Biological Process Categories. Figure showing a joined Gene Ontology graph providing an overview of the relationship between the 39 Gene Ontology Biological Process (GO‐BP) categories that were identified from the output of the SVM as having a precision‐at‐40 value equal or larger than 0.75, as indicated in the highlighted polygonal nodes. [file 1756-0381-6-8-S1.tiff]
